# Effect of Carboxylmethyl Cellulose Coating and Osmotic Dehydration on Freeze Drying Kinetics of Apple Slices

**DOI:** 10.3390/foods2020170

**Published:** 2013-05-21

**Authors:** Jamshid Rahimi, Ashutosh Singh, Peter Olusola Adewale, Akinbode A. Adedeji, Michael O. Ngadi, Vijaya Raghavan

**Affiliations:** Department of Bioresource Engineering, McGill University, 21111 Rue Lakeshore, Sainte-Anne-de-Bellevue, Quebec, H9X 3V9, Canada; E-Mails: jamshid.rahimi@mail.mcgill.ca (J.R.); peter.adewale@mail.mcgill.ca (P.O.A.); akinbode.adedeji@mail.mcgill.ca (A.A.A.); michael.ngadi@mcgill.ca (M.O.N.); vijaya.raghavan@mcgill.ca (V.R.)

**Keywords:** apple slices, osmo-dehydration, freeze drying, carboxyl methyl cellulose coating, drying kinetics

## Abstract

The effect of different concentrations of sugar solution (hypertonic) (30%, 45% and 60% w/v) and carboxyl methyl cellulose (CMC) (0%, 1% and 2% w/v) coating on freeze drying of apple slices was studied. In total, nine treatments with respect to concentrations of hypertonic solution and coating layer were prepared to analyze their influence on the physical and chemical properties of freeze dried apple slices. It was observed that increase in the sugar solution concentration, decreased the moisture content of the apple slices significantly impacting its water activity, texture and sugar gain. Application of different concentrations of CMC coating had no significant effect on the properties of dried apple slices. A significant change was observed for color of CMC coated freeze dried apple slices pretreated with 60% sugar solution. Drying kinetics of pretreated apple slices were fitted by using two drying models, Newton’s and Page’s. Page’s model showed higher *R*-square and lower root mean square error (RSME) compared to Newton’s model.

## 1. Introduction

Osmotic dehydration of food materials includes partial removal of water when they are immersed in a hypertonic solution for a particular time and solution temperature. During this process, water transfers from the product to the osmotic solution and the osmotic solute, such as salt and sugar, diffuse from the medium into the immersed product [1]. Osmotic dehydration has been widely used as a pretreatment to conventional drying techniques as it preserves the natural flavor, color and texture during the drying process and improves the nutritional, sensorial and functional properties of the food [2,3]. In an ideal osmotic condition, a semi-permeable membrane is permeated by the solvent molecules, but the solute molecules cannot penetrate through it. Since the cell membranes of vegetables and fruits are alive and can expand under the effect of growing turgor pressure, the solvent molecules, and also part of the solute molecules, freely pass through these cellular membranes. Some leakage of solutes such as sugars, organic acids, salts, minerals have been reported [4]. During osmotic dehydration solute, like sugar, gets absorbed from the osmotic solution into the food material and results in changes in the textural and organoleptic qualities of the food product. Due to this undesirable solid gain in osmotically dehydrated food materials, use of osmotic dehydration by food industries is limited [5] and it is important to find a way to solve this problem. One practical method to restrict undesirable solid gain is by coating samples with edible materials such as gum Arabic, carboxylmethyl cellulose and xanthan gum before osmotic dehydration [5]. Some researchers have suggested that to obtain a proper osmo-dehydration, edible coatings should possess some characteristic features such as good mechanical strength, desirable sensory properties, ability to quickly form film, high water diffusivity and low solute diffusivity and also high stability [6]. Ali *et al.* [7] in 2010 used gum Arabic to enhance the shelf life and post harvest quality of tomato fruit. They found that gum Arabic coating had a remarkable delay in the change of weight, firmness, soluble solids concentration and color during storage compared to uncoated tomatoes. In another study, use of carboxylmethyl cellulose coating on apple slices before osmotic dehydration had higher water loss and lower solids uptake [1]. 

In recent years, a dramatic change in consumer’s attitude towards processed food products has been observed. It is now important that fruit and vegetable products have high nutritional and organoleptic qualities. Food industries and most researchers are interested in finding ways to produce high value dehydrated food products [8]. It is well known that freeze dried foods maintain desirable properties such as original color, good taste, retained aroma, and excellent rehydration behavior. Freeze drying causes the least damage on the texture of the dried products and as such can be used as a substitute to other methods of drying such as hot air, vacuum, microwave, and osmotic dehydration [9]. This drying process involves primarily freezing the food samples in a freezer, followed by keeping them under low pressure condition with the use of sufficient heat for ice sublimation [10]. The low temperature and vacuum condition, the degradation of vitamins and loss of aroma are significantly limited. In 2010 Agnieszka and Andrezej studied the structural impact of osmotically pretreated freeze dried strawberries on their mechanical properties and reported that osmotic dehydration can limit the shrinkage of freeze dried strawberries. They also found that this process strengthened the fruit structure by increasing their cell wall thickness [11]. 

In this study the effect of carboxylmethyl cellulose coating prior to osmotic dehydration pretreatment on physical and chemical properties of freeze dried apple slices was evaluated. 

## 2. Experimental Section

### 2.1. Materials

Apples (cv. Red Delicious) were purchased from a local supermarket and then stored at 5 °C under 80%–90% humidity for 2 days prior to experimental studies. Sucrose was used as the solute for preparation of hypertonic solution. Ascorbic acid powder obtained from Fisher, Fair Lawn, NJ, USA was used to prepare 1% ascorbic acid solution to prevent enzymatic browning in the cut apple slices. Carboxylmethyl cellulose (CMC, TIC Pretested^®^ Ticalose^®^ CMC 2500 C Powder, TIC Gums, Inc., Belcamp, MD, USA) was used as the edible coating for the apple slices.

### 2.2. Carboxylmethyl Cellulose Coating

Apples were washed, peeled and cut uniformly into slices of length 20 mm, width of 20 mm, and thickness of 5 mm. Slices were immediately dipped in ascorbic acid solution to prevent enzymatic browning. Two different concentrations of CMC coating (1% and 2%, w/v) were prepared. Non-coated (0% CMC) apple slices were used as control. To prepare the CMC solution, distilled water was boiled in a microwave, the CMC powder was added to boiled water, stirred, and heated again in microwave for 3 min. Apple slices were dipped in CMC solutions (1% and 2%) at ambient temperature for 30 s; the surface was dried using filter paper for a few minutes followed by oven drying at 70 °C for 10 min.

### 2.3. Osmotic Dehydration Pre-Treatment

Sucrose powder was used to prepare three different concentrations (30%, 45% and 60%, w/v) of osmotic solution. To prepare osmo-dehydrated apple slices with and without CMC coating, slices were dipped in sugar solutions at 45 °C for 90 min. The product/solution ratio was maintained at 1:10 (weight basis). Slices were removed from the solution and their surface was dried with a filter paper for few minutes.

### 2.4. Freeze Drying Procedure

After pretreatment, the osmo-dehydrated samples were kept in a freezer (Thermo Forma, Model 5698 REL#11, Marietta, OH, USA) at −80 °C for 24 h; then they were dried in a freeze dryer (Thermo Savant, Model: MODULYOD-115, Holbrook, NY, USA) at −50 °C and 150 mbar pressure for 24 h. To obtain drying rate curve, the samples were weighed at 2 h interval during freeze drying and at the end of freeze drying, the samples were kept in a desiccator before carrying out further analysis. 

A total of nine treatments of osmo-dehydrated apple slice were prepared: (1) 0% carboxylmethyl cellulose coating and 30% sugar solution (*0C30S*); (2) 0% carboxylmethyl cellulose coating and 45% sugar solution (*0C45S*); (3) 0% carboxylmethyl cellulose coating and 60% sugar solution (*0C60S*); (4) 1% carboxylmethyl cellulose coating and 30% sugar solution (*1C30S*); (5) 1% carboxylmethyl cellulose coating and 45% sugar solution (*1C45S*); (6) 1% carboxylmethyl cellulose coating and 60% sugar solution (*1C60S*); (7) 2% carboxylmethyl cellulose coating and 30% sugar solution (*2C30S*); (8) 2% carboxylmethyl cellulose coating and 45% sugar solution (*2C45S*); (9) 2% carboxylmethyl cellulose coating and 60% sugar solution (*2C60S*).

### 2.5. Drying Kinetic Models

The initial moisture content of apple slices was estimated using gravimetric method. Apple slice were weighed (10 g), and then dried at 105 °C to a constant weight in a forced air convection oven (Isotemp 700, Fisher Scientific, Pittsburgh, PA, USA). The weight of apple slices before and after oven drying was used to measure the moisture content. Moisture loss ratio (MLR) was measured to monitor the drying behavior of osmo-dehydrated apple slices during 24 h of freeze drying. The MLR was calculated using Equation (1):

MLR = (M_0_ − M)/M_e_ (1)

Where, M_0_ is the moisture content of apple slices after soaking in the hypertonic solution and before freeze drying, M is the moisture content at any time during freeze drying, and M_e_ is the equilibrium moisture content. The moisture content at the end of freeze drying was considered as the equilibrium moisture content. Modified Newton’s (Equation (2)), and Page’s (Equation (3)) models were used to describe moisture loss during freeze drying.

MLR = [1 − exp(−k × *t*)] (2)

MLR = [1 − exp(−k × *t*^2^)] (3)

Where, k is the model constant, *i.e.*, moisture loss rate constant and *t* is the drying time. In order to choose the better suitable model for fitting the freeze drying kinetics, the value of *R*-Square (*R*^2^) and the root mean square error (RMSE) associated with the prediction models were taken into account. The kinetic parameters were obtained using nonlinear regression in MATLAB (Version 7.6.0.324 R2008a, The Mathworks, Inc., Natick, MA, USA).

### 2.6. Sugar Gain

The purpose of coating apple slices is to obstruct the penetration of solute (sugar solution) inside the food material, with minimal effect on the rate of water removal [1]. Therefore, it is important to measure the sugar uptake during soaking. Sugar gain (SU) by apple slices was obtained by using:

SU = (S*_t_* − S_0_)/S*_t_* × 100 (4)

Where, S*_t_* is solid content after soaking, and S_0_ is solid content before soaking and after coating.

### 2.7. Shrinkage

Food products undergo thickness and volumetric changes due to water loss during drying process, which is defined as shrinkage. These changes occur continuously during drying and affect the physical properties, as well as the transport phenomena. Thus, shrinkage has an important influence on the drying rate and on the texture of dried fruits and vegetables [12]. To measure shrinkage, thickness of fresh and freeze dried samples were measured by electronic digital caliper (Marathon Watch CO Ltd., Richmond Hill, ON, Canada), Shrinkage (S) was calculated using the equation:

S = [(T_0_ − T*_t_*)/T_0_] × 100 (5)

Where, T_0_ is the initial thickness and T*_t_* is thickness of the samples after freeze drying.

### 2.8. Textural Properties

The mechanical behavior may be interpreted by some certain physical parameters measured through a puncture test carried out on an Instron Universal Testing Machine (Model 4502, Instron, Canton, MA, USA). A cylindrical 3.8 mm diameter probe was used to puncture the samples at a crosshead speed of 25 mm/min until the entire sample was penetrated. The required energy (J) to reach breaking point and maximum force required to puncture the dried apple slices were used as parameters to determine the textural properties.

### 2.9. Water Activity

Water activity measurement of freeze dried samples was carried out with the AQUA LAB (Long Sault, ON, Canada) at 22.9 ± 0.1 °C.

### 2.10. Color Measurements

After freeze drying, color property of apple slices were obtained by using Konica Minolta Colorimeter (Model No: CR-300, Konika Minolta, Tokyo, Japan). The CIE 1976 (*L* (whiteness or brightness/darkness), *a* (redness/greenness), and *b* (yellowness/blueness)) color space were the three color measurements determined for all the samples. The instrument was calibrated before experiments with a white ceramic plate (*Y* = 93.35, *X* = 0.3152, *Z* = 0.3212). The total color change (ΔE) was calculated to describe the color change during the drying process [13].

ΔE = [(*L*_0_ − *L_t_*)^2^ + (*a*_0_ − *a_t_*)^2 ^+ (*b*_0_ − *b_t_*)^2^]^1/2^ (6)

Where, *L*_0_, *a*_0_, and *b*_0_ are the initial color measurements of fresh apple slices and *L_t_*, *a_t_*, and *b_t_* were the color measurements after freeze drying.

### 2.11. Statistical Analysis

All experiments were conducted with three replicates and mean values were reported. SAS software (SAS 9.2, SAS Institute Inc., Cary, NC, USA) was used to perform the ANOVA procedure for all the data. Student Newman Kelus (SNK) method was used to estimate significant differences among the means at a 5% probability level. 

## 3. Results and Discussion

### 3.1. Drying Behavior

**Figure 1:**
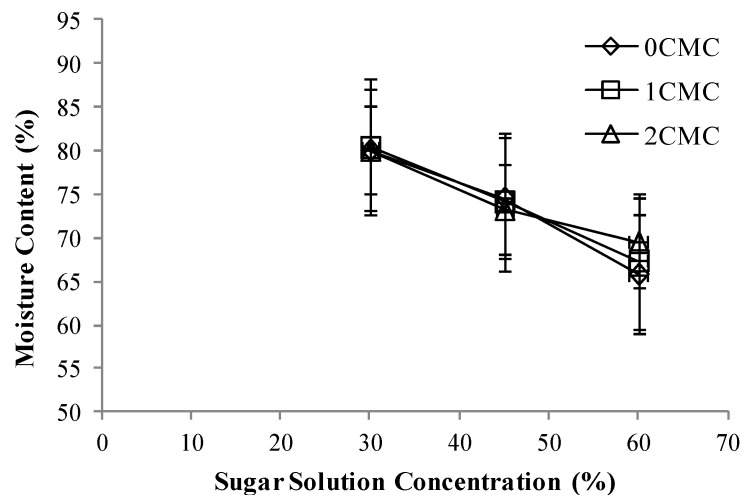
Moisture content at different concentrations of carboxylmethyl cellulose (CMC) coating (0%, 1% and 2%) *vs.* sugar solution concentrations, *p* < 0.05.

**Figure 2:**
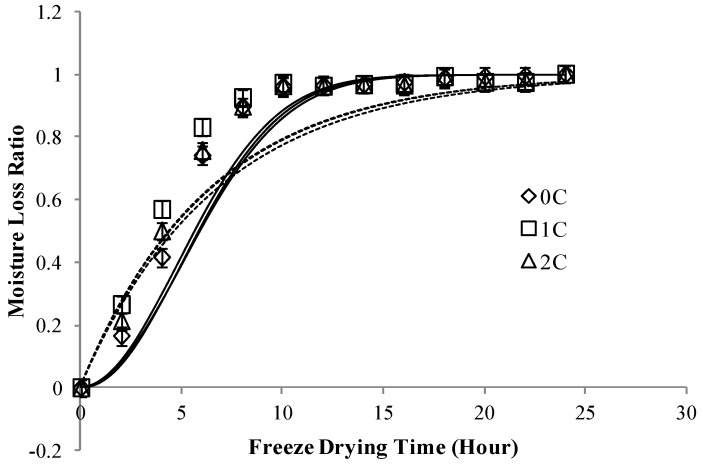
Moisture loss ratio (MLR*) of freeze dried apple slices coated with different concentarions of CMC, *p* < 0.05. Note: Continuous lines, predicted values via the Page’s model; Dash lines, predicted values via the Newton’s model.

**Figure 3:**
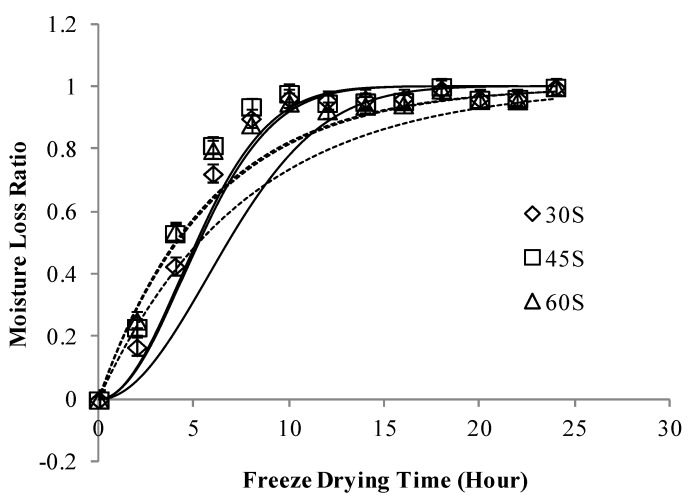
Moisture loss ratio (MLR*) of freeze dried apple slices pre-treated in different concentarions of sugar solution), *p* < 0.05. Note: Continuous lines, predicted values via the Page’s model; dash lines, predicted values via the Newton’s model.

It was found that different concentrations of CMC coating did not have any significant effect on the moisture content and moisture loss ratio (Figure 1, Figure 2, Figure 3), while, by increasing the concentration of sugar solution, it notably decreased the moisture content. It can be observed from Figure 1, the moisture content of apple slices treated in 60% sugar solution is less than 30% and 40% sugar solutions. Apple samples with different concentrations of CMC coating that were osmo-dehydrated under similar concentrations of sugar solution, had similar moisture content. Our results were in agreement with the work, conducted by Falade *et al.* in 2003 [14], they reported a decrease in instantaneous moisture content of cashew apples by increasing osmotic solution concentration. They explained that higher solution concentration promoted greater water-solute transfer between the osmotic medium and apple slices. Similar results were also obtained by Falade *et al.* in 2004 [15] in their study on the adsorption isotherms of osmo-oven dried African star apples. They reported that higher the pretreatment sucrose solution concentration, the lower was the moisture content of the samples.

Figure 2, Figure 3 show moisture loss ratio (MLR*) of freeze dried apple slices coated with different concentrations of CMC and slices pre-treated in different concentrations of sugar solution, respectively. It was observed that after around 10 h of freeze drying, all samples reached a constant rate drying state and there was no significant change in moisture loss ratio. Menlik *et al*. [16] in 2010 in their study on freeze drying of apples reported that drying rate of apple decreased during freeze drying. Our results were in agreement with their observation on change of falling rate to constant rate of drying state after a few hours of freeze drying.

From Figure 2 it can be observed that there is a difference in the predicted and experimental values, this can be attributed to the loss of moisture during oven drying for 10 min at 70 °C of apple slices after osmotic dehydration. But the kinetic results from Newton’s and Page’s drying models (Table 1) strongly suggest that CMC coating can decrease the kinetic of moisture loss. This phenomenon can be attributed to the fact that CMC layer created a barrier and prevented moisture loss during freeze drying. Apple slices osmo-dehydrated in less concentrated hypertonic solutions had more moisture content and showed more potential for moisture loss than apple slices dipped in higher concentrated hypertonic solutions. From Table 1 it can be observed that the freeze drying kinetics of apple slices is better fitted by the Page’s model than the Newton’s model because higher the *R*-square and lower the root mean square error (RSME), better the fitness of drying model. 

**Table 1:** Parameters of first-order kinetic model of moisture loss for different pre-treatments of apple slices.

Model						
Treatment	Newton			Page		
	K	*R*-Square	RMSE	K	*R*-Square	RMSE
*0C*	0.1579	0.9312	0.0977	0.0216	0.9993	0.0101
*1C*	0.1560	0.9400	0.0902	0.0214	0.9987	0.0135
*2C*	0.1489	0.8990	0.1240	0.0197	0.9918	0.0354
*30S*	0.1313	0.9037	0.1214	0.0150	0.9966	0.0229
*45S*	0.1681	0.9135	0.0112	0.0249	0.9951	0.0266
*60S*	0.1725	0.9367	0.0918	0.0262	0.9986	0.0138
Average		0.9207	0.0894		0.9967	0.0204

*0C*, 0% carboxyl methyl cellulose coating; *1C*, 1% carboxyl methyl cellulose coating; *2C*, 2% carboxyl methyl cellulose coating; *30S*, 30% sugar solution; *45S*, 45% sugar solution; *60S*, 60% sugar solution; The treatments report the average of the experimental conditions with respect to CMC and sugar concentrations.

### 3.2. Sugar Gain

**Figure 4:**
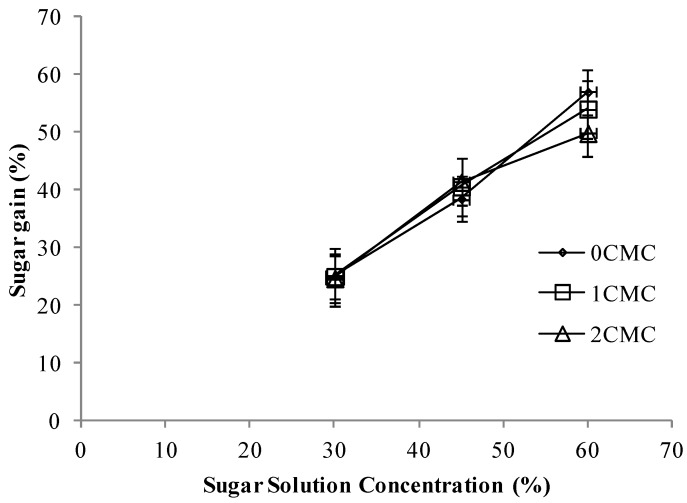
Sugar gain at different concentrations of CMC coating *vs.* sugar solution concentrations, *p* < 0.05.

The purpose of coating the apple slices was to decrease the sugar gain during osmo-dehydration. The three different CMC coatings had no significant effect on the sugar gain during osmotic dehydration (Figure 4). It is worthwhile to mention the effect of sugar solution concentration; it was observed that higher sugar solution induced higher sugar content in the apple dried by osmo-dehydration. In a similar study on osmotic dehydration of apple cylinders by Li *et al.* [17] in 2006, the authors reported that by increasing the concentration of sugar solution the solid gain increased. In another study [14], it was found that apple slices immersed in a sugar solution with 68° Brix had higher sugar content than apple slices soaked in 60° Brix and those of 60° Brix had significantly higher sugar content than the samples osmodehydrated in 52° Brix sugar solution.

### 3.3. Shrinkage Thickness

If water is lost during the drying process from food materials, they usually undergo volumetric changes, which affect the drying rate and textural properties. During the drying of apple slices this phenomenon occurs continuously and can affect their physical properties. Decreasing the shrinkage of dried fruits is possible by covering them with a coating material [12]. 

**Figure 5:**
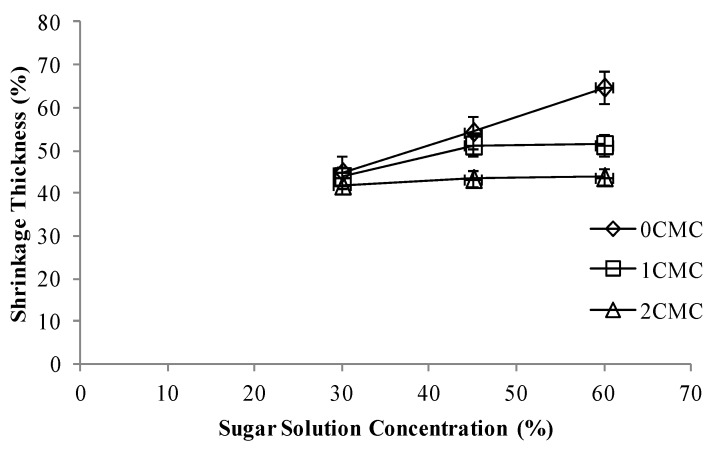
Shrinkage thickness at different concentrations of CMC coating *vs.* sugar solution concentrations.

Figure 5 represents the effect of CMC coating on the shrinkage thickness of apple slices at all three concentrations of sugar solutions. Sugar solution concentration had no significant effect on the shrinkage thickness whereas more CMC coating a sample received the less it shrunk. Our results were in agreement with those obtained by Lenart and Piotrowski [18]. They reported that the coated dried apples had less shrinkage than the uncoated samples regardless of the type of substance used for coating [18]. For non CMC coated apple slices, the shrinkage thickness increased with increase in the concentration of hypertonic solution. As per observed results osmotic dehydration had no significant effect on the shrinkage thickness because after osmotic treatment the apple slices were dried at 70 °C for 10 min, this might have lead to activation of pectin methyl esterase (PME), which may make the texture of apple slices firmer [19,20]. In a study conducted on osmotic pretreatment of two varieties of strawberries Viberg *et al.* (1998) [20] observed that when the pretreated strawberries were thermally processed and stored in 60% sucrose solution for three days the strawberries neither shrunk nor swelled, they attributed this to activation of (PME). Our result on action of osmotic pretreatment were in accordance with results obtained by Changrue *et al.* (2008) [19], where they observed that osmotically pretreated strawberries had the lowest percentage change in volume when compared to samples without osmotic pretreatment.

### 3.4. Textural Properties

**Figure 6:**
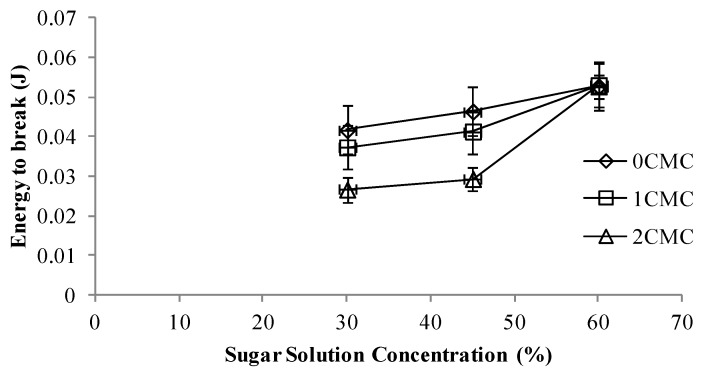
Required energy to break at different concentrations of CMC coating *vs.* sugar solution concentration.

**Figure 7:**
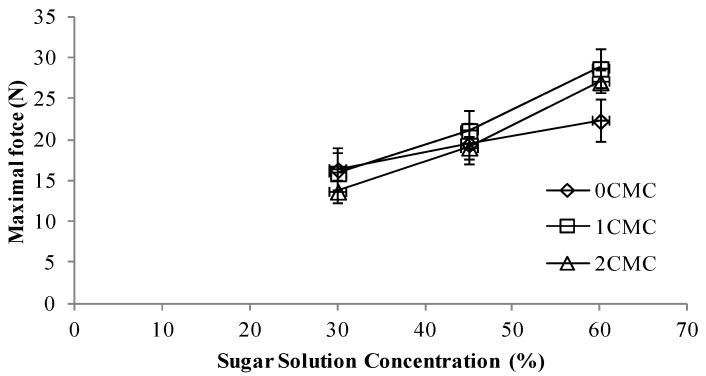
Maximum force at different concentrations of CMC coating *vs.* sugar solution concentrations

Firmness is one of the most important textural attributes in determining the quality of dried apple slices especially from the consumer satisfaction point of view. To study the firmness of the samples, Instron Universal Testing Machine was used. Energy to break (J) was not statistically significant (*p* < 0.05) and there was no correlation among the different concentrations of CMC coating and the energy required to break the sample (Figure 6). The energy to break increased with an increase in sugar solution concentration and for each particular sugar solution; increasing the concentration of CMC coating decreased the required energy to break. Hence it can be concluded that by increasing the sugar solution concentration and decreasing the CMC coating, the final product obtained was firmer compared to control. Similar observation was obtained for the maximal force (N) required to break the sample. Increase in sugar content of the hypertonic solution led to an increase in the force required to break the sample (Figure 7).

### 3.5. Water Activity

**Figure 8:**
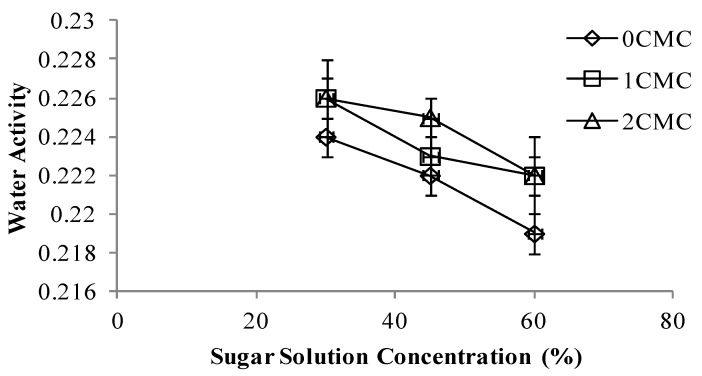
Water activity at different concentrations of CMC coating *vs.* sugar solution concentrations.

Increasing the concentration of hypertonic solution decreased the water activity of the apple slices. Non-coated apple slices had the lowest water activity under all sugar solution concentrations due to higher moisture loss moisture during freeze drying. Slices coated with 1% and 2% CMC had no significant difference in their water activity under 30% and 60% sugar concentration, while in solution with 45% sugar apple slices coated with 1% CMC had lower water activity (Figure 8).

### 3.6. Color Properties

**Figure 9:**
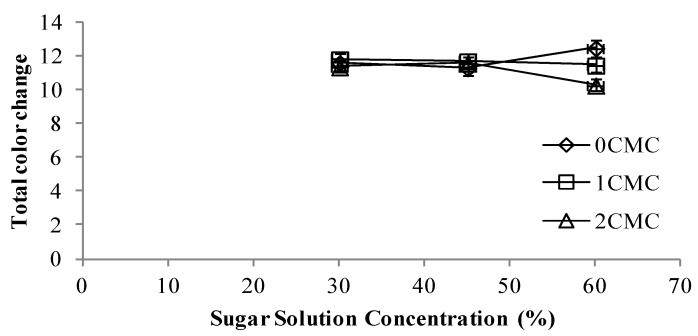
Total color change at different concentrations of CMC coating *vs.* sugar solution concentrations.

One of the most important quality factors of food, which plays an important role in consumer acceptance is its color [21]. From Figure 9 it can be observed that CMC coating had no significant effect on the total color change for free dried apple slices dipped in 30% and 45% sugar solutions. Whereas, samples coated with 2% CMC and osmo dehydrated in 60% sugar solution had the lowest total color change and the non-coated sample had the highest color change.

## 4. Conclusions

This study revealed that the sugar solution concentration had major effects on moisture content, water activity, textural properties, and shrinkage of the samples. As the concentration of the hypertonic solutions increased, the moisture content and water activity decreased significantly. Higher sugar solution concentration showed higher shrinkage, maximal force and energy to break. While considering the amount of CMC used for coating, there was no significant difference found for most of the physicochemical properties. From the study it was observed that the drying rate reached constant rate after about 10 h of freeze drying. It was also concluded that the Page’s model is a better model than Newton’s model to study the freeze drying behavior of CMC coated, osmo dehydrated apple slices.

## References

[1] Dehghannya J., Emam-Djomeh Z., Sotudeh-Gharebagh R., Ngadi M. (2006). Osmotic dehydration of apple slices with carboxy-methyl cellulose coating. Dry. Technol..

[2] Singh B., Panesar P.S., Nanda V., Kennedy J.F. (2010). Optimisation of osmotic dehydration process of carrot cubes in mixtures of sucrose and sodium chloride solutions. Food Chem..

[3] Amami E., Khezami L., Jemai A.B., Vorobiev E. (2012). Osmotic dehydration of some agro-food tissue pre-treated by pulsed electric field: Impact of impeller’s Reynolds number on mass transfer and color. J. King Saud Univ. Eng. Sci..

[4] Torreggiani D. (1993). Osmotic dehydration in fruit and vegetable processing. Food Res. Int..

[5] Khin M.M., Zhou W., Perera C.O. (2006). A study of the mass transfer in osmotic dehydration of coated potato cubes. J. Food Eng..

[6] Singh C., Sharma H.K., Sarkar B.C. (2010). Influence of process conditions on the mass transfer during osmotic dehyration of coated pineapple samples. J. Food Proc. Preserv..

[7] Ali A., Maqbool M., Ramachandran S., Alderson P.G. (2010). Gum arabic as a novel edible coating for enhancing shelf-life and improving postharvest quality of tomato (*Solanum lycopersicum* L.) fruit. Postharvest Biol. Technol..

[8] Huang L.-I., Zhang M., Mujumdar A.S., Sun D.-F., Tan G.-W., Tang S. (2009). Studies on decreasing energy consumption for a freeze-drying process of apple slices. Dry. Technol..

[9] Cui Z.-W., Li C.-Y., Song C.-F., Song Y. (2008). Combined microwave-vacuum and freeze drying of carrot and apple chips. Dry. Technol..

[10] Hammami C., René F., Marin M. (1999). Process-quality optimization of the vacuum freeze-drying of apple slices by the response surface method. Int. J. Food Sci. Technol..

[11] Agnieszka C., Andrzej L. (2010). Structural impact of osmotically pretreated freeze-dried strawberries on their mechanical properties. Int. J. Food Prop..

[12] Schultz E.L., Mazzuco M.M., Machado R.A.F., Bolzan A., Quadri M.B., Quadri M.G.N. (2007). Effect of pre-treatments on drying, density and shrinkage of apple slices. J. Food Eng..

[13] Dadali G., Apar D.K., Özbek B. (2007). Color change kinetics of okra undergoing microwave drying. Dry. Technol..

[14] Falade K.O., Akinwale T.O., Adedokun O.O. (2003). Effect of drying methods on osmotically dehydrated cashew apples. Eur. Food Res. Technol..

[15] Falade K.O., Aworh O.C. (2004). Adsorption isotherms of osmo-oven dried african star apple (*Chrysophyllum albidum*) and african mango (*Irvingia gabonensis*) slices. Eur. Food Res. Technol..

[16] Menlik T., Özdemir M.B., Kirmaci V. (2010). Determination of freeze-drying behaviors of apples by artificial neural network. Expert Syst. Appl..

[17] Li H., Ramaswamy H.S. (2006). Osmotic dehydration of apple cylinders: I. Conventional batch processing conditions. Dry. Technol..

[18] Lenart A., Piotrowski D. (2001). Drying characteristics of osmotically dehydrated fruits coated with semipermeable edible films. Dry. Technol..

[19] Changrue V., Orsat V., Raghavan G.S.V. (2008). Osmotically dehydrated microwave-vacuum drying of strawberries. J. Food Proc. Preserv..

[20] Viberg U., Freuler S., Gekas V., Sjöholm I. (1998). Osmotic pretreatment of strawberries and shrinkage effects. J. Food Eng..

[21] Maskan M. (2001). Kinetics of colour change of kiwifruits during hot air and microwave drying. J. Food Eng..

